# Supplementary material for: Comparison of Tumour-Specific Phenotypes in Human Primary and Expandable Pancreatic Cancer Cell Lines
Source: Int J Mol Sci. 2023 Aug 31;24(17):13530. doi: 10.3390/ijms241713530 (PMC10488093; doi:10.3390/ijms241713530)
Supplement: Supplementary file 1 [file ijms-24-13530-s001.zip › ijms-2498856-supplementary.pdf]

Supplementary data

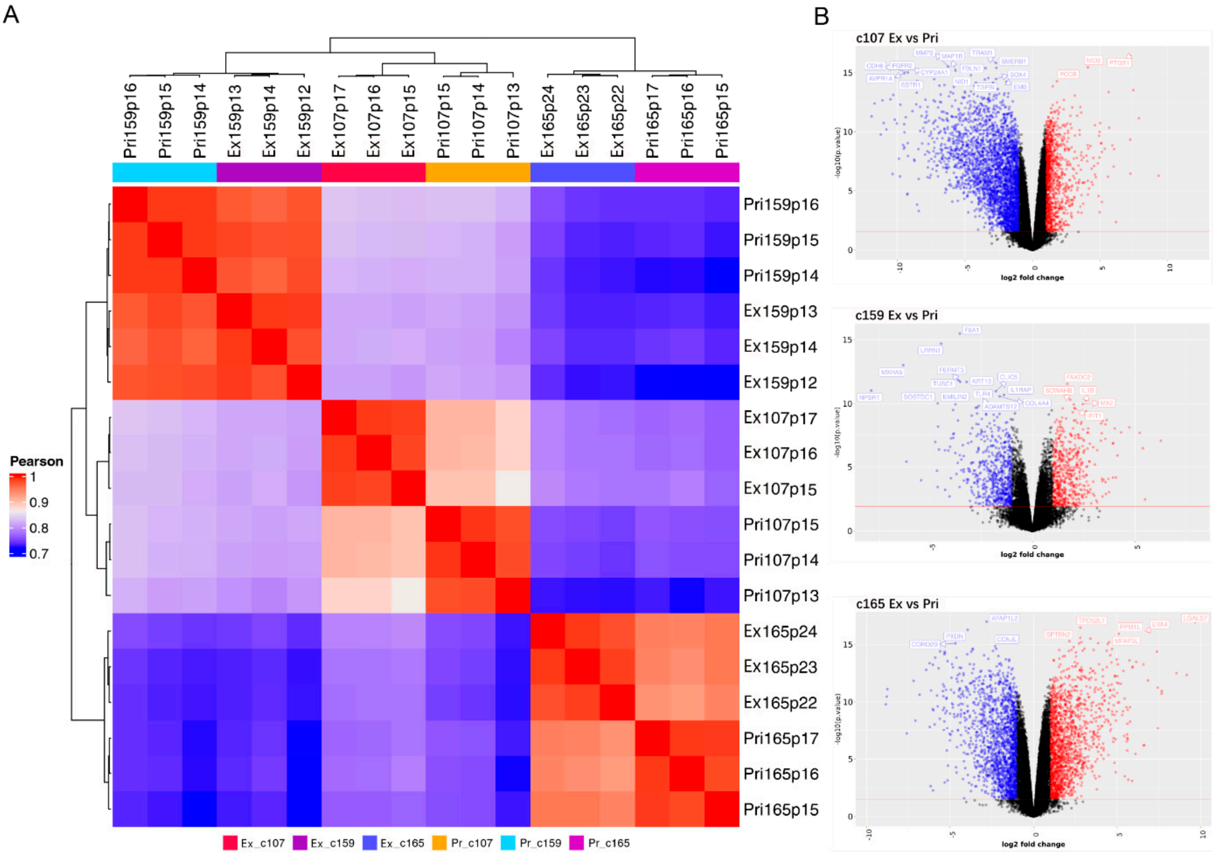

Figure S1. Cluster analysis and Volcano plots (expandable samples vs primary samples). A. Cluster analysis. Pearson correlation heat map illustrating the correlation between the primary and expandable samples of each cell line from three sequential passages. B. Volcano plots labelling top significantly DEGs. The genes with a log2 fold change were significant. The upregulated and downregulated genes were centralized towards the right and left sides, respectively. The red line below each volcano plot represented the threshold of significances.

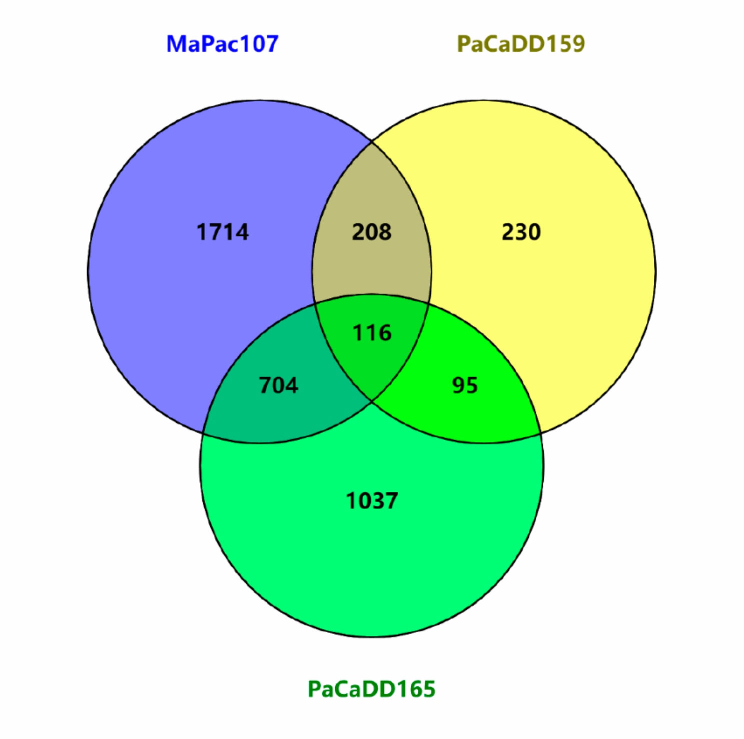

Figure S2. Venn diagram. DEGs were selected with logFC ＞1.5 or ＜-1.5; adjusted p-value＜0.05 among the mRNA expression of MaPac107, PaCaDD159, and PaCaDD165 (expandable samples vs primary samples).

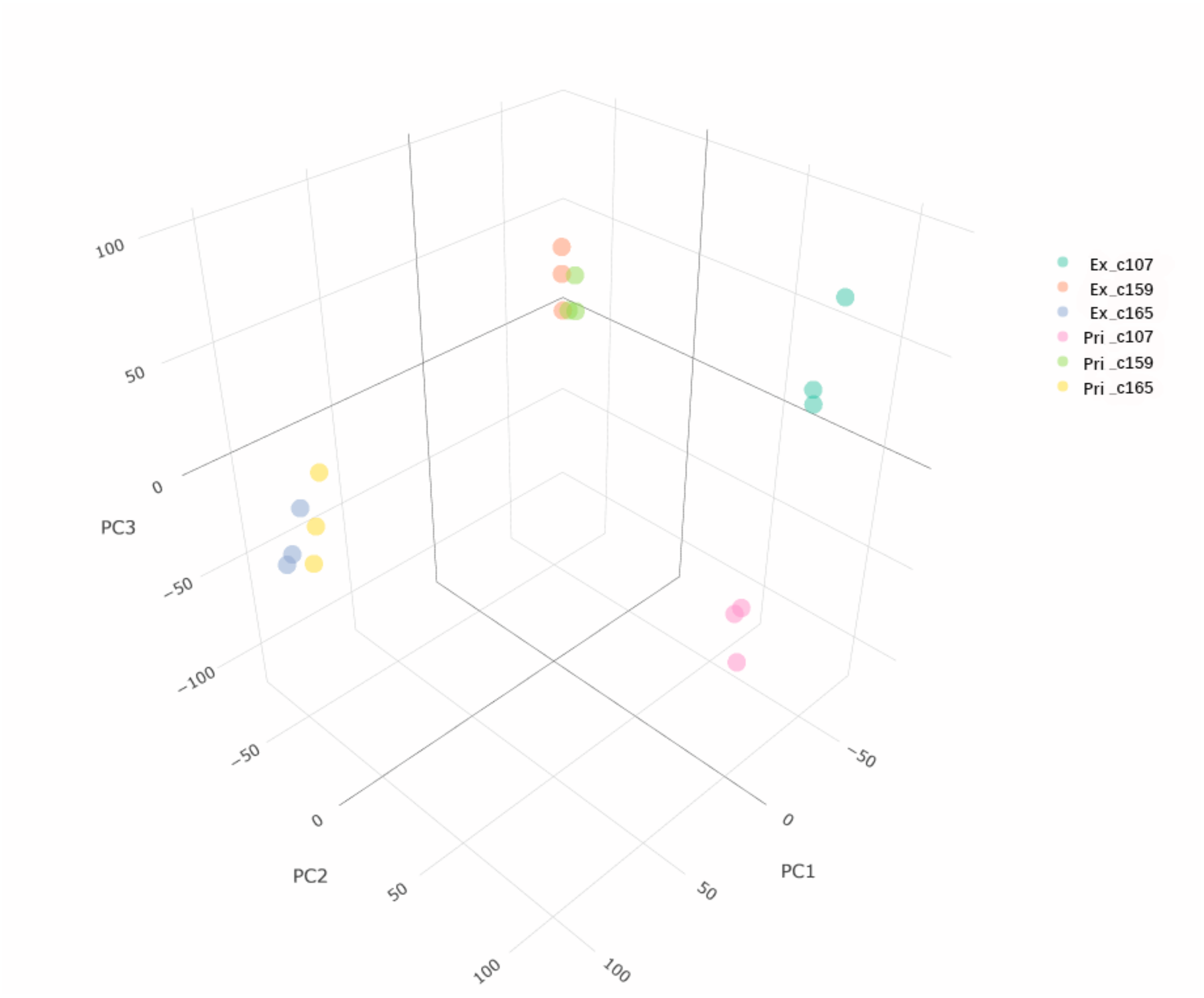

Figure S3. 3D PCA map depicting the distribution pattern of the dots of primary and expandable samples of each cell line.

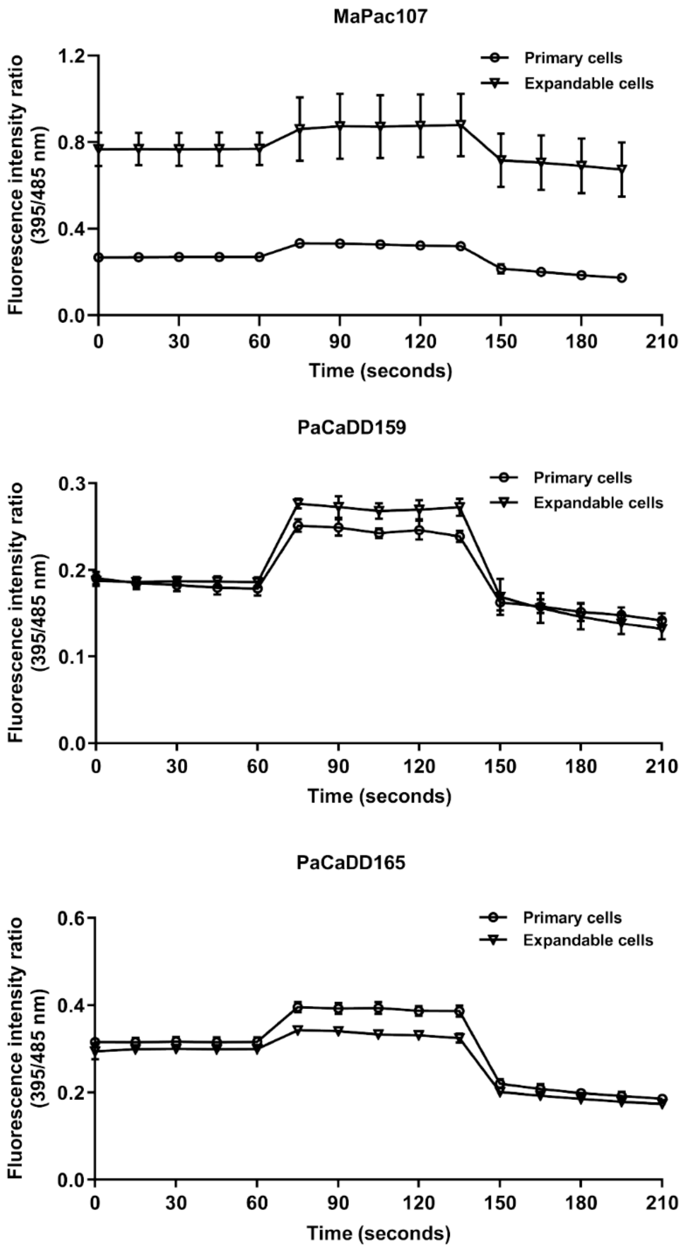

Figure S4. The dynamics of E_{GSH} on primary and expandable pancreatic cancer cell lines expressing Grx1-roGFP3+. Cells were treated with H_2O_2 and DTT. Fluorescence intensity was measured by a scheduled program of the SPARK Plate Reader.

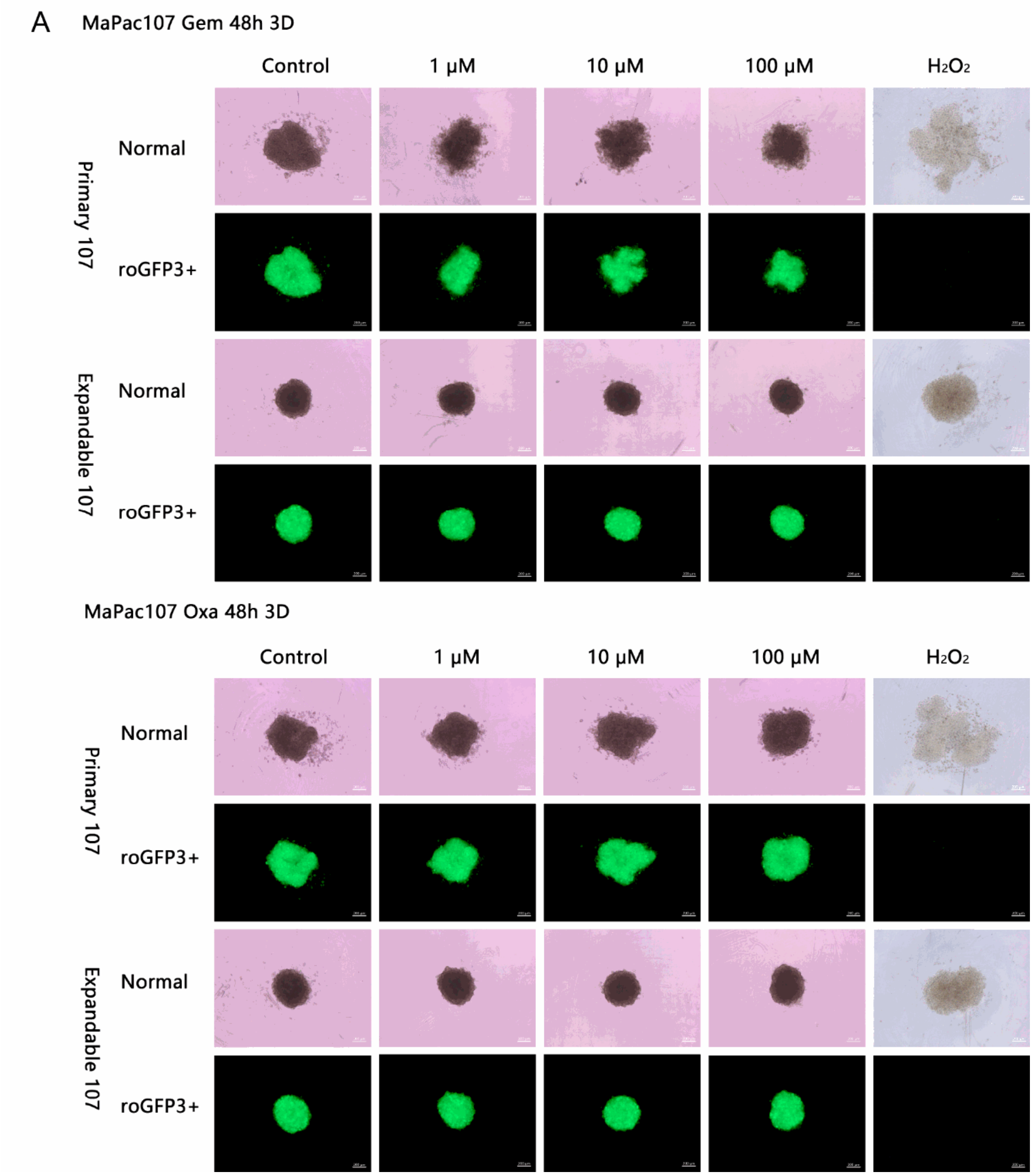
A
MaPac107 Gem 48h 3D
Control
1 μM
10 μM
100 μM
H2O2
Primary 107
Normal
roGFP3+
Expandable 107
Normal
roGFP3+
MaPac107 Oxa 48h 3D
Control
1 μM
10 μM
100 μM
H2O2
Primary 107
Normal
roGFP3+
Expandable 107
Normal
roGFP3+

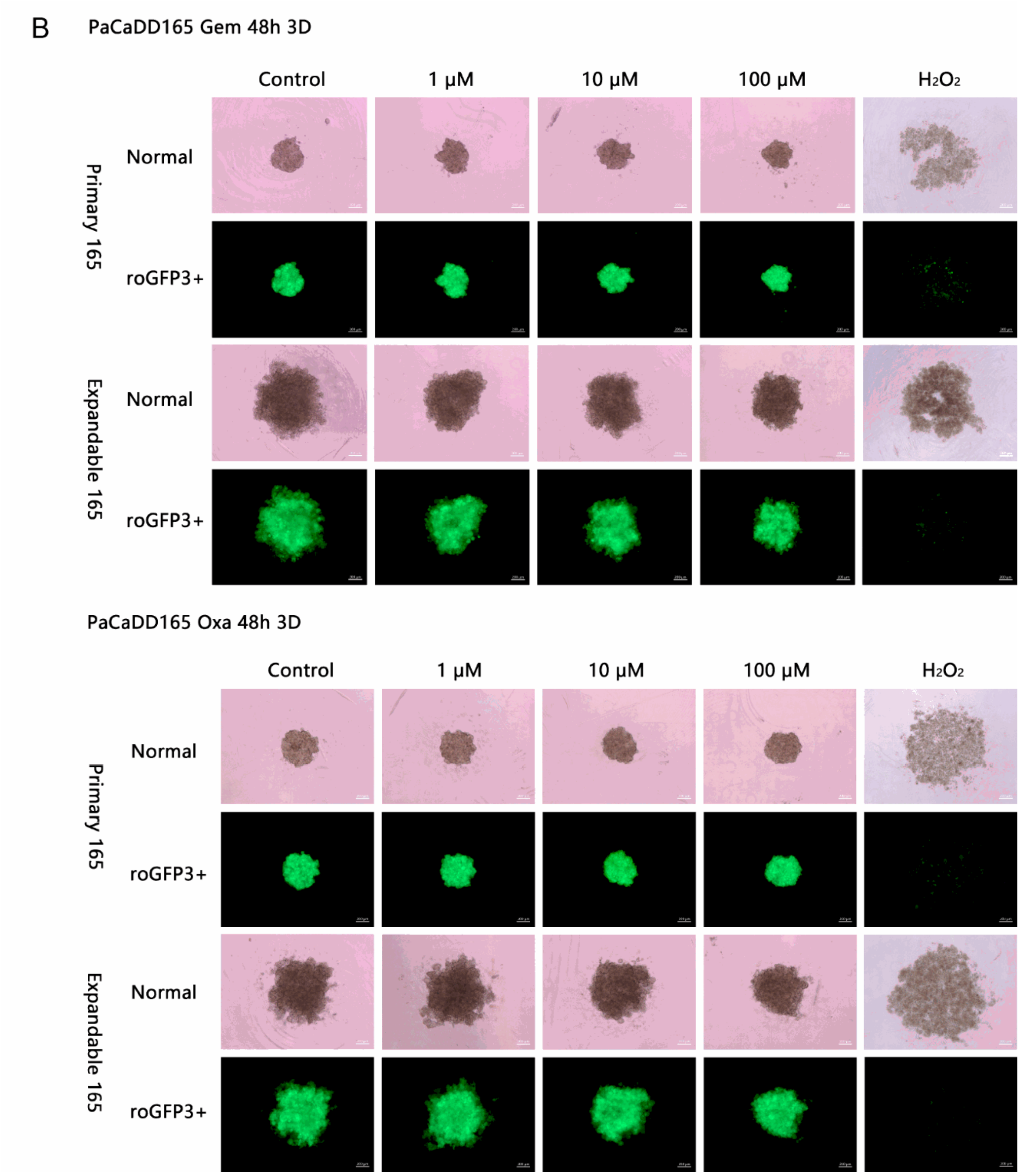

Figure S5. Cellular status in primary and expandable pancreatic cancer cell lines expressing Grx1-roGFP3+ in 3D culture after incubation with gemcitabine and oxaliplatin. Representative pictures were derived using Carl Zeiss Axio Vert. A1 microscope under 5X magnification. Scale bar was 200 μm. A. MaPac107. B. PaCaDD165.

Target Chemotherapy Drugs	
OXALIPLATIN	ETOPOSIDE
GEMCITABINE	METHOTREXATE
IRINOTECAN	VINBLASTINE
TEMOZOLOMIDE	CETUXIMAB
PROCARBAZINE	IDARUBICIN
SUNITINIB	SN-38
DAUNORUBICIN	DOXORUBICIN

Table S1. Fourteen target chemotherapy drugs were selected from 94 potential target drugs concurrently shared by MaPac107, PaCaDD159, and PaCaDD165. SN-38 was 7-ETHYL-10-HYDROXYCAMPTOTHECIN.

Gemcitabine	Oxaliplatin
1 mg/mL	5 mg/mL
100 µg/mL	1 mg/mL
10 µg/mL	300 µg/mL
1 µg/mL	30 µg/mL
100 ng/mL	3 µg/mL
10 ng/mL	300 ng/mL
1 ng/mL	30 ng/mL
100 pg/mL	3 ng/mL
10 pg/mL	300 pg/mL
1 pg/mL	30 pg/mL
100 fg/mL	-
10 fg/mL	-
1 fg/mL	-
0.1 fg/mL	-

Table S2. Different concentrations of gemcitabine and oxaliplatin used for the IC_{50} assay.

Cell types	Drugs	IC_{50} 48 h (µM)	IC_{50} 72 h (µM)
Primary MaPaC107	Gemcitabine	0.3	0.2
Expandable MaPaC107	Gemcitabine	0.27	0.12
Primary MaPaC107	Oxaliplatin	445	259
Expandable MaPaC107	Oxaliplatin	277	216
Primary PaCaDD159	Gemcitabine	0.021	0.016
Expandable PaCaDD159	Gemcitabine	0.23	0.098
Primary PaCaDD159	Oxaliplatin	32.5	36.2
Expandable PaCaDD159	Oxaliplatin	214	127
Primary PaCaDD165	Gemcitabine	0.19	0.067
Expandable PaCaDD165	Gemcitabine	0.056	0.043
Primary PaCaDD165	Oxaliplatin	95.8	39.2
Expandable PaCaDD165	Oxaliplatin	94.5	51.1

Table S3. IC_{50} values.

Cell type	Symbol	Related Drugs	Type of gene	Chromosome	LogFC	Adjusted p-value
MaPac107	ABCG2	Gemcitabine	Protein coding	chr4	5.64096093	2.44288E-11
	SLC28A3		Protein coding	chr9	-3.611762	9.06347E-05
	HCP5		lncRNA	chr6	-3.8040031	3.39835E-07
	KDR		Protein coding	chr4	-4.6177598	6.2399E-11
	ABCG2	Oxaliplatin	Protein coding	chr4	5.64096093	2.44288E-11
	PTGS2		Protein coding	chr1	-4.371547	1.15679E-07
	PARD3B		Protein coding	chr2	-5.513123	4.77848E-07
	CXCL10		Protein coding	chr4	-5.5576791	9.58309E-06
PaCaDD159	TNF	Gemcitabine	Protein coding	chr6	4.32344247	3.37643E-06
	CXCL10	Oxaliplatin	Protein coding	chr4	3.92419323	1.38162E-05
PaCaDD165	IGF2	Gemcitabine	Protein coding	chr11	-3.1825554	4.01533E-11
	DKK1	Oxaliplatin	Protein coding	chr10	-5.3181536	2.36644E-12

Table S4. DEGs related to gemcitabine and oxaliplatin were expressed by MaPac107, PaCaDD159, and PaCaDD165 (expandable samples versus primary samples). The values of logFC were > 3 or < -3; the adjusted p-values were < 0.05.

Cell lines comparison

Comparisons between groups of Ex/Pri cells were performed with analysis of Welchs's two sample t-test using Matlab R2022b. A value of $P < 0.05$ was considered statistically significant.

The indicative behavioral trait of the groups that was compared was the chemosensitivity of primary and expandable pancreatic cancer cell lines with the hypothesis that Ex and Pri cell lines behave accordingly. Measurement data is represented by target drug concentration x_M and an indicator y_M for surviving cells after a given time. There are k data points in each group. Groups are defined by cell line (MaPac107, PaCaDD159, PaCaDD165), treatment time (48 h and 72 h) and target drugs (gemcitabine and oxaliplatin).

Group 1<br>MaPac107 (Ex/Pri),<br>oxaliplatin, 48 h	Group 4<br>MaPac107 (Ex/Pri),<br>oxaliplatin, 72 h	Group 7<br>MaPac107 (Ex/Pri),<br>gemcitabine, 48 h	Group 10<br>MaPac107 (Ex/Pri),<br>gemcitabine, 72 h
Group 2<br>PaCaDD159 (Ex/Pri),<br>oxaliplatin, 48 h	Group 5<br>PaCaDD159 (Ex/Pri),<br>oxaliplatin, 72 h	Group 8<br>PaCaDD159 (Ex/Pri),<br>gemcitabine, 48 h	Group 11<br>PaCaDD159 (Ex/Pri),<br>gemcitabine, 72 h
Group 3<br>PaCaDD165 (Ex/Pri),<br>oxaliplatin, 48 h	Group 6<br>PaCaDD165 (Ex/Pri),<br>oxaliplatin, 72 h	Group 9<br>PaCaDD165 (Ex/Pri),<br>gemcitabine, 48 h	Group 12<br>PaCaDD165 (Ex/Pri),<br>gemcitabine, 72 h

Table S5. Groups definitions.

To compare the behavioral traits, Welch's two-sample t-test is used with the hypothesis that for a given group Ex and Pri cell lines show the same behavior. Welch's two-sample t-test [1] is a parametric test that compares mean and standard deviation of two independent data samples $\{x_{M1}, y_{M1}\}$ and $\{x_{M2}, y_{M2}\}$. The objective is to proof whether their underlying relationship is the same. The null hypothesis for Welch's two-sample t-test is that the models do expose the same behavior with a significance level of 5%. Following procedure is proposed.

1. Control and normalization of the measurement data:
   - compensation of offset from raw data with mean value
   $y_{M,Ex} = y_{M,Ex,raw} - \bar{y}_{M,Ex,raw}$ and $y_{M,Pri} = y_{M,Pri,raw} - \bar{y}_{M,Pri,raw}$
   - use of the $x_{M,j} = -\log\left(d_{M,j}\right)$ with $d_{M,j}$ the drug concentration in g/l.
   - outliers have to be removed, cf. [2] and below for M_L

$$\left|\frac{y_{M,j} - M_L(x_{M,j})}{\sigma_L(x_{M,j})}\right| < 5$$

2. Regression for a model $f_{regr}(\beta)$ on all data points of the group's intersect $\{x_{M,Ex}, y_{M,Ex}\} \cap \{x_{M,Pri}, y_{M,Pri}\}$ or use of local mean M_L (cf. below).
   $f_{regr}(\beta)$ is a model for the cell lines' behavior.
3. Calculation of the residuals $R_{Ex} = y_{M,Ex} - f_{regr}(\beta, x_{M,Ex})$ and $R_{Pri} = y_{M,Pri} - f_{regr}(\beta, x_{M,Pri})$
   Both residuals are vectors that contain all values of the group.
   Alternatively residuals $R_{Ex} = y_{M,Ex} - M_L(x_{M,Ex})$ and $R_{Pri} = y_{M,Pri} - M_L(x_{M,Pri})$
4. The test statistic is

$$t = \frac{\bar{R}_{Ex} - \bar{R}_{Pri}}{\sqrt{\frac{{\sigma_{REx}}^2}{k_{\mathrm{Ex}}} + \frac{{\sigma_{Pri}}^2}{k_{\mathrm{Pri}}}}}$$

   $\bar{R}_i$ being the mean of R_i, σ_{Ri} being the sample standard deviations of R_i, k_i being the respective sample sizes.
5. In the case where it is not assumed that the two data samples are from populations with equal variances, the test statistic under the null hypothesis has an approximate Student's t distribution with a number of degrees of freedom DOF given by Satterthwaite's approximation.

$$DOF = \frac{\left(\frac{\sigma_{REx}^{2}}{k_{Ex}} + \frac{\sigma_{RPri}^{2}}{k_{Pri}}\right)^{2}}{\frac{1}{k_{Ex}-1}\left(\frac{\sigma_{REx}^{2}}{k_{Ex}}\right)^{2} + \frac{1}{k_{Pri}-1}\left(\frac{\sigma_{RPri}^{2}}{k_{Pri}}\right)^{2}}$$

6. Test the null hypothesis. If the test is not rejected, both residuals expose the same behavior. Because the residuals were calculated on an identical basis (f_{regr} or M_L) it is deduced that the cell lines behaviors are identical, too.

New tools for local statistical behavior

For the statistical comparation new tools to define local properties in any desired point $x \in [S^1, S^2]$ were developed. The approach is similar to the use of Savitzky-Golay filters [3]. Given the assumption that the measurement data can be represented by a continuous model, one can deduce that there is an influence of the measuring points $\{x_{M,i+\varepsilon}, y_{M,i+\varepsilon}\}$ close to $x_{M,i}$ on the models' value $f_{Regr}(\beta_i, x_{M,i})$. The influence of the neighboring points declines with distance - a basic assumption in differential calculus. The method is presented here with one input variable but can be expanded to more variables.

In an intuitive approach it is assumed that the influencing horizon fades away with distance in terms of a normalized gaussian relationship. The factor Λ defines how strong the neighbors weigh in to the local value.

$$influence \sim \frac{1}{\Lambda\sqrt{2\pi}} e^{-\frac{1}{2}\left(\frac{x_{M,j}-x}{\Lambda}\right)^{2}}$$

The maximum distance between neighboring measuring points (sampling) limits the details of the model (Nyquist-Shannon-Theorem). For coarsely sampled measurement data a high model order does not make sense. The maximum sampling distance of the measuring points is taken as a minimum value for Λ. The value is iteratively increase in a way that the local point density $D_L(x)$ (cf. below) is at no place lower than three. The best choice of the value for Λ should be examined in further works.

Local Number of Points

The local number of points gives an estimate how many data points are close to x. The integral of $n(x)$ is equal to the number of measurement points. x is not excusively a part of the measurement values and can be freely chosen.

$$n(x) = \sum_{j=1}^{k} 1 \cdot \frac{1}{\Lambda\sqrt{2\pi}} e^{-\frac{1}{2}\left(\frac{x_{M,j}-x}{\Lambda}\right)^{2}} = \sum_{j=1}^{k} 1 \cdot r_j(x)$$

The integral of the normalized Gauß function is 1, which is necessary to yield accurate approximations of the local statistics.

$$\int_{-\infty}^{+\infty} r_j(x)\, dx = 1$$

Therefore, we can replace the expression above in $n(x)$ and get

$$\int_{-\infty}^{+\infty} n(x)\, dx = \int_{-\infty}^{+\infty} \left[\sum_{j=1}^{k} 1 \cdot r_j(x)\right] dx = \left[\int_{-\infty}^{+\infty} r_j(x)\, dx\right]\left[\sum_{j=1}^{k} 1\right] = 1 \cdot k$$

The integral of the local number of points equals the number of points!

$$\int_{-\infty}^{+\infty} n(\vec{x})\, dx = k$$

The formulas can be adapted to higher dimensions of the input variable $x \epsilon \mathbb{R}^n$. For the local number of points this is shown

$$n(\vec{x}) = \sum_{j=1}^{k} 1 \cdot \frac{1}{\vec{\Lambda} \cdot \frac{\left(\vec{x}_{M,j} - \vec{x}\right)^T}{\left\|\vec{x}_{M,j} - \vec{x}\right\|} \sqrt{2\pi}} e^{-\frac{1}{2}\left(\frac{\left\|\vec{x}_{M,j} - \vec{x}\right\|}{\vec{\Lambda} \cdot \frac{\left(\vec{x}_{M,j} - \vec{x}\right)^T}{\left\|\vec{x}_{M,j} - \vec{x}\right\|}}\right)^2} = \sum_{i=1}^{n} 1 \cdot r_i(\vec{x})$$

$$\vec{\Lambda} = \begin{pmatrix} \Lambda_1 \\ \vdots \\ \Lambda_n \end{pmatrix}$$

Local Density

The local density gives an estimate how many data points are close to x, in relation to all data points.

$$D_L(x) = \frac{1}{k} \sum_{j=1}^{k} 1 \cdot \frac{1}{\Lambda\sqrt{2\pi}} e^{-\frac{1}{2}\left(\frac{\vec{x}_{M,j} - \vec{x}}{\Lambda}\right)^2} = \frac{1}{k} \sum_{i=1}^{k} 1 \cdot r_j(x)$$

Local Mean

The local mean at every point x represents the highest (local) probability for the normally distributed measured data. Plotted over x, is most close to the 'true' models' behavior. It can be calculated numerically, only.

$$M_L(x) = \frac{\sum_{j=1}^{k} y_j \cdot r_j(x)}{n(x)}$$

Local standard deviation

The local standard deviation shows trends of the spread of the disturbance function over the observation range.

$$\sigma_L(x) = \frac{\sum_{j=1}^{k} \sqrt{\left[r_j(x) \cdot \left(y_j - M_L(x_j)\right)\right]^2}}{n(x)}$$

With the local standard deviation $\sigma_L(x)$ the probability that $f_{Regr}\left(x_{M,j}\right)$ is the "true model" can be calculated via the Chi-square test.

Standard deviation indicator

The local standard deviation can be summarized in one indicator, as a global indicator for the spread of the data.

$$\sigma_I = \frac{\sum_{j=1}^{k} \sigma_L\left(x_j\right)}{n}$$

Exact coefficient of determination (R_Σ-Value)

The use of the coefficient of determination (R-square) is widely seen critically in literature [2]. Assuming the local mean to be the "true" value of the model, an adapted definition for a more significant coefficient of determination is given.

$$R_\Sigma = \frac{\sum_{j=1}^{k} \sqrt{\left[y_j - M_L\left(x_j\right)\right]^2}}{\sum_{j=1}^{k} \sqrt{\left[y_j - f_{Regr}\left(x_j\right)\right]^2}}$$

Error in input variables

There is an error both in the input $\sigma_y(x)$ and output $\sigma_x(x)$ variable. There is an intuitive, iterative approach to calculate an estimate of the input error. The regression models' derivative (steepness) $\nabla f_{Regr}(x)$ can be calculated.

$$y\left(x_j\right) \cong f_{Regr}\left(\beta, x_j\right) + \nabla f_{Regr}\left(\beta, x_j\right) \cdot \sigma_x\left(x_j\right) + \sigma_y\left(x_j\right)$$

The error functions are approximated with two unknown parameters, σ_x and σ_y that are assumed constant over the observation range.

$$\sigma_x(x) = \sigma_x = const.$$
$$\sigma_y(\mathrm{x}) = \sigma_y = const.$$

By regressions, the estimators for σ_x and σ_y can be found

$$min \sum_{j=1}^{k} \left(y - f_{Regr}(\mathrm{x}_j) - \nabla f(x_j) \cdot \sigma_x - \sigma_y\right)^2$$

This procedure works well only with regression models that have varying slope (no straight lines). The borders of the observation range must be excluded, as the slope of $f_{Regr}(\vec{x})$ cannot assumed be correct lack of neighboring points. The reduced observation range is

$$[x_{min} + \Lambda, x_{max} - \Lambda]$$

Control of measurement data

The measurement data is controlled for spread and corrected for offset. The values of y_M lie in the range [0,1]. Global variance σ_I is approx. 10%, comparable to the calculated variance σ_y in the output variable (7%). It is interesting that the part of variance triggered by the input variable σ_x is higher (21%). This indicates that the process of diluting the drug might be an important factor for variances.

Variance σ_I for gemcitabine and oxaliplatin are nearly equal (10% and 11% respectively).

Variance σ_I for time 48h and 72h are nearly equal (10% and 11% respectively).

Variance σ_I for cell lines are nearly equal (10% to 11%).

Group	σ_I	σ_y	σ_x
MaPac107_Gem_48h_IC	0.09	0.05	0.18
MaPac107_Gem_48h_PC	0.09	0.05	0.12
MaPac107_Gem_72h_IC	0.11	0.06	0.28
MaPac107_Gem_72h_PC	0.12	0.06	0.25
MaPac107_Oxa_48h_IC	0.09	0.05	0.37
MaPac107_Oxa_48h_PC	0.09	0.05	0.27
MaPac107_Oxa_72h_IC	0.11	0.04	0.5
MaPac107_Oxa_72h_PC	0.1	0.06	0.35
PaCaDD159_Gem_48h_IC	0.09	0.08	-0.03
PaCaDD159_Gem_48h_PC	0.13	0.11	0.08
PaCaDD159_Gem_72h_IC	0.08	0.05	0.25
PaCaDD159_Gem_72h_PC	0.09	0.09	-0.05
PaCaDD159_Oxa_48h_IC	0.11	0.05	0.53
PaCaDD159_Oxa_48h_PC	0.09	0.06	0.2
PaCaDD159_Oxa_72h_IC	0.15	0.1	0.32
PaCaDD159_Oxa_72h_PC	0.13	0.09	0.31
PaCaDD165_Gem_48h_IC	0.12	0.08	0.07
PaCaDD165_Gem_48h_PC	0.1	0.07	0.17
PaCaDD165_Gem_72h_IC	0.11	0.07	0.1
PaCaDD165_Gem_72h_PC	0.07	0.03	0.12
PaCaDD165_Oxa_48h_IC	0.16	0.13	0.11
PaCaDD165_Oxa_48h_PC	0.09	0.07	0,01
PaCaDD165_Oxa_72h_IC	0.12	0.04	0.52

PaCaDD165_Oxa_72h_PC	0.09	0.08	0.09

Table S6. Raw data analysis for each group and its elements (Ex and Pri cells) measurement data.

For all groups, less than 1% of the data points were identified as outliers and were removed from the data sets.

Group	Outlier [%]
MaPac107_Gem_48h_IC/PC	0.00%
MaPac107_Gem_72h_IC/PC	0.00%
MaPac107_Oxa_48h_IC/PC	0.28%
MaPac107_Oxa_72h_IC/PC	0.28%
PaCaDD159_Gem_48h_IC/PC	0.00%
PaCaDD159_Gem_72h_IC/PC	0.00%
PaCaDD159_Oxa_48h_IC/PC	0.28%
PaCaDD159_Oxa_72h_IC/PC	0.00%
PaCaDD165_Gem_48h_IC/PC	0.27%
PaCaDD165_Gem_72h_IC/PC	0.97%
PaCaDD165_Oxa_48h_IC/PC	0.28%
PaCaDD165_Oxa_72h_IC/PC	0.56%

Table S7. Outliers overview for each group.

Choice of regression model

For the clinical IC_{50} test, nonlinear models ('sigmoids') are used to identify the point of 50% inhibition, which is defined by the saddle point of the fitted sigmoid. The so-called dose-response curves' model is not revealed by the producers of the clinical test, so for this paper, Gompertz, Logistics and Hill equation have been tested. The coefficient of determination (R-square or R_Σ) was used to estimate the goodness-of-fit. It was found that they are inappropriate for a comparison of cells behavior, because of their reduced complexity. For clinical every day analyses sigmoids meet the needs, their advantage being to indicate the one important value, the inhibition point.

Gompertz Function

$$f_{Gompertz}(z) = \beta_1 \cdot e^{[-\beta_2 \cdot e^{[-\beta_3 \cdot z]}]}$$

A transformation is necessary:

$$z = -x$$

$$IC_{50,Gompertz} = \frac{1}{\beta_3} \log\left[\frac{-\log\left[\frac{1}{2}\right]}{\beta_2}\right]$$

Logistic S-Function

$$f_{Logistic}(z) = \frac{\beta_1}{1 + \frac{\beta_2}{\beta_3 - 1} e^{[-\beta_1 \beta_2 \cdot z]}}$$

A transformation is necessary:

$$z = -x + x_{max}$$

$$IC_{50,Logistics} = \frac{\log\left[\frac{\frac{2\beta_1}{\beta_1 + \beta_3} - 1}{\frac{\beta_2}{\beta_3} - 1}\right] - 1}{\beta_1 \cdot \beta_2} + x_{max}$$

Hill's Equation

$$f_{Hill}(z) = \beta_1 + \frac{\beta_2 - \beta_1}{1 + \left[\frac{-z}{\beta_3}\right]^{\beta_4}}$$

A transformation is necessary:

$$z = -x + x_{max}$$

$$IC_{50,Hill} = -\frac{\beta_2 - 1}{1^{\frac{1}{\beta_3}}} + x_{max}$$

Finite power Series polynomials

Finite power series polynomials were also tested on the measurement data

$$f_{power}(z) = \sum_{i=0}^{o-1} \beta_i z^i$$

The best fitting order $o - 1$ of the power series is determined with the Bayesian information criterion in the form presented in [4].

$$BIC = k \cdot \ln\left(\frac{SS_{Res}}{k}\right) + o \cdot \ln(k)$$

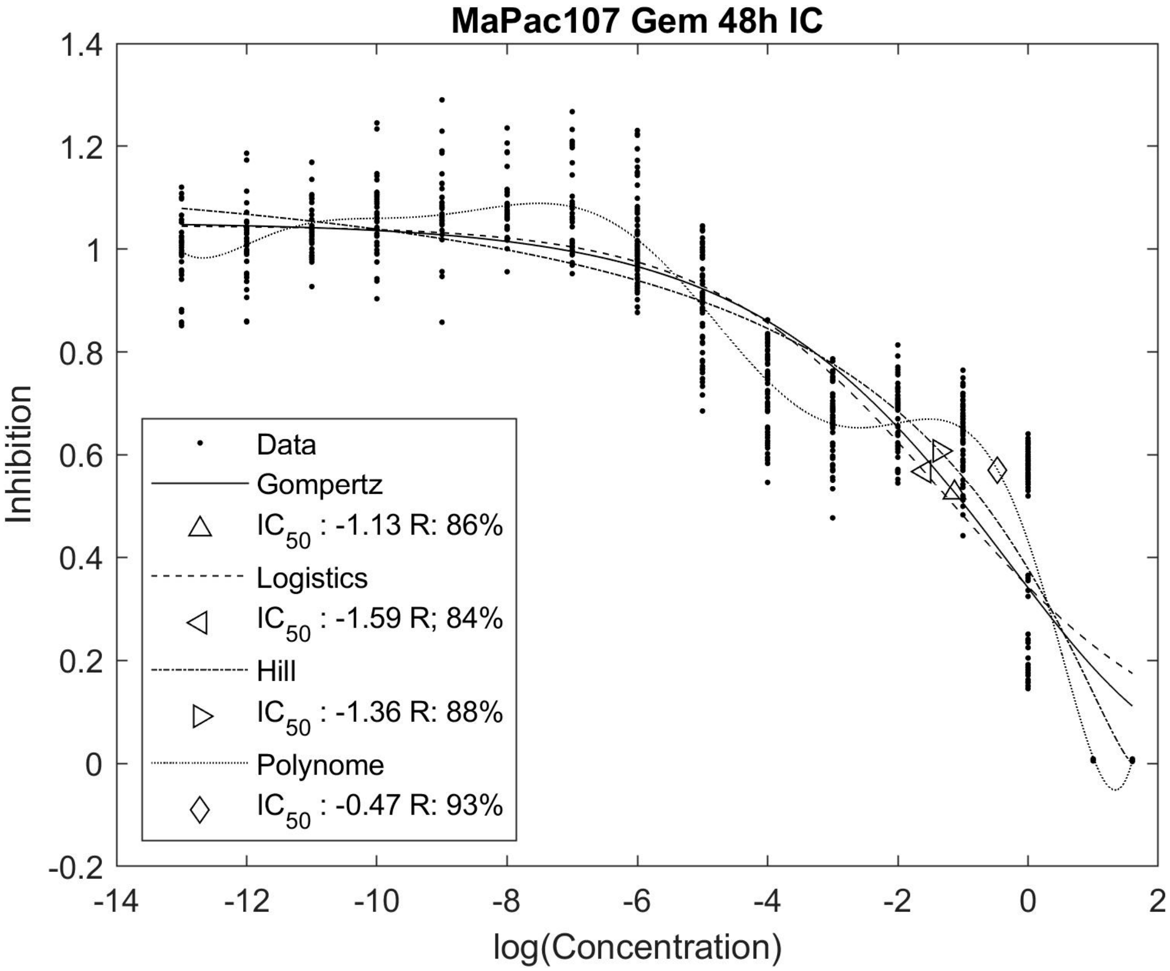

Figure S6. Example for diverse regression models, quality indicator coefficient of determination; IC_{50} values calculated on the turning point of the curves.

Choice of regression model

The average values for all cell lines are shown in table below. The finite power series polynomials have a significantly better R_Σ value and are therefore chosen for the comparison of the cell lines' behavior.

R_Σ-Value	Gompertz	Hill	Logistics	Polynome
MaPac107 (all)	88%	88%	88%	92%
PaCaDD159 (all)	88%	87%	88%	90%
PaCaDD165 (all)	86%	85%	86%	91%
All	87%	87%	87%	91%

Table S8. Exact coefficient of determination in dependence of cell line for sigmoids and polynome models.

An interesting fact that has to be interpreted is that the Oxa inhibitor shows higher R_Σ values for sigmoids than the Gem inhibitor. This indicates the cells' behavior exposed to the inhibitor matches more the smooth behavior of a sigmoid.

R_Σ-Value	Gompertz	Hill	Logistics	Polynome	Overall
Gem	84.6%	84.1%	85.3%	91.6%	86.4%
Oxa	89.8%	89.8%	89.6%	90.4%	89.9%

Table S9. Exact coefficient of determination in dependance of the inhibitor for sigmoids and polynome models.

The IC_{50} value (turning point) of the polynomials was found numerically. The IC_{50} value is found to be lower for the Gem inhibitor than for the Oxa inhibitor.

IC_{50} Value	Gompertz	Hill	Logistics	Polynome	Overall
Gem	-2.93	-3.60	-3.11	-3.45	-3.27
Oxa	-1.46	-1.45	-1.59	-1.58	-1.52

Table S10. IC_{50} value in dependence of the inhibitor for sigmoid and polynome models.

Results of Welchs' two-sample t-test

Welch's two-sample t-test was performed on all groups of intersect data of Pri and Ex cells with the null hypothesis that behavior is identical and with a significance level of 5%. The null hypothesis was not rejected for all groups. Therefore, the behavior of the Ex and Pri cell lines has been judged as identical with a probability of 95%. The findings were identical for residuals of regression model f_{regr} and local mean M_L.

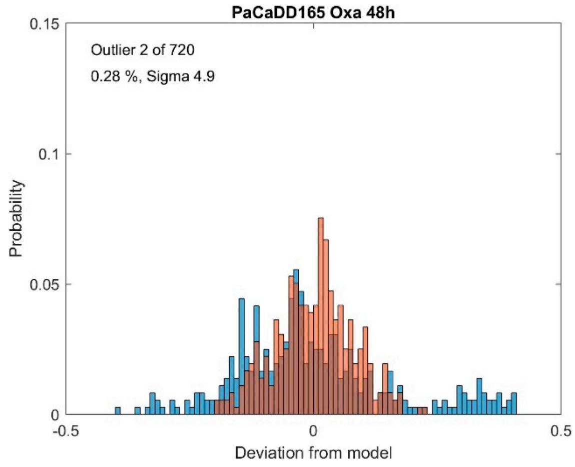

Histogram of residual for regression model f_{regr}

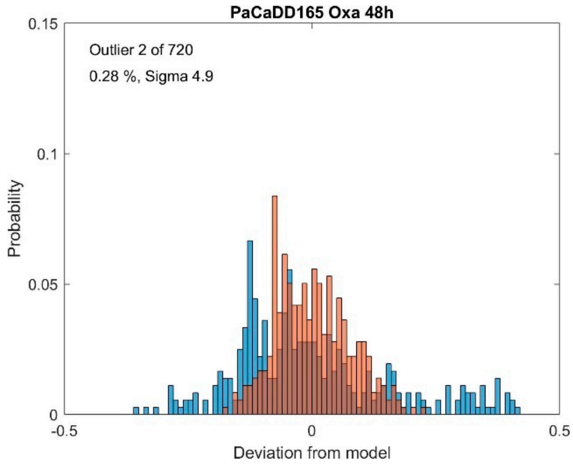

Histogram of residual of local mean M_L

Figure S7. Example for the results of the t-test for residuals calculated with regression model f_{regr} and local mean M_L.

References

1. Brandt, S. *Datenanalyse: Mit Statist. Methoden u. Computerprogrammen*, 2nd ed.; Bibliographisches Institut: Mannheim: Wien, Austria, 1975; ISBN 978-3-411-01488-0.
2. Fahrmeir, L.; Kneib, T.; Lang, S. *Regression: Modelle, Methoden und Anwendungen*; Statistik und ihre Anwendungen; Springer: Berlin/Heidelberg, Germany, 2007; ISBN 978-3-540-33932-8.
3. Crimins, F. Numerical Recipes in C++: The Art of Scientific Computing: 2nd Ed.; W. Press, S. Teykolsky, W. Vetterling, and B. Flannery; Pub Date: February 2002; ISBN: 0-521-75033-4; 1064 pp.; $70.00 (Hardcover). *Appl. Biochem. Biotechnol.* **2003**, *104*, 95–96. https://doi.org/10.1007/s12010-003-0001-6.
4. Schneider, M. *Datenanalyse für Naturwissenschaftler, Mediziner und Ingenieure*; Springer Spektrum: Berlin/Heidelberg, Germany, 2020; ISBN 978-3-662-61866-0.
